# Effects of Algicidal Macrophyte Metabolites on Cyanobacteria, Microcystins, Other Plankton, and Fish in Microcosms

**DOI:** 10.3390/toxins15090529

**Published:** 2023-08-28

**Authors:** Svetlana Kurbatova, Nadezhda Berezina, Andrey Sharov, Ekaterina Chernova, Evgeny Kurashov, Yulia Krylova, Igor Yershov, Alexander Mavrin, Natalia Otyukova, Elena Borisovskaya, Roman Fedorov

**Affiliations:** 1 Papanin Institute for Biology of Inland Waters, Russian Academy of Sciences, 152742 Borok, Russia; kurb@ibiw.ru (S.K.); sharov_AN@mail.ru (A.S.); evgeny_kurashov@mail.ru (E.K.); juliakrylova@mail.ru (Y.K.);; 2 Zoological Institute, Russian Academy of Sciences, 199034 St. Petersburg, Russia; 3 St. Petersburg Federal Research Center, Russian Academy of Sciences, 199178 St. Petersburg, Russia; s3561389@ya.ru

**Keywords:** cyanotoxins, *Microcystis*, *Aphanizomenon*, *Dolichospermum*, macrophyte allelochemicals, phytoplankton, zooplankton, medaka, nutrient excretion, grazing

## Abstract

To control harmful algae blooms (HABs), methods based on natural mechanisms are now required. We investigated the effects of an algicide derived from macrophyte metabolites, namely mixtures of gallic, tetradecanoic, heptanoic, and octanoic acids (1:1:1:1 mass ratio, a total concentration of 14 mg/L), on the biomass of cyanobacteria and other plankton and the production of microcystins under experimental conditions. Two types of microcosms have been created: simple (microalgae, cyanobacteria, and zooplankton) and complex (microalgae, cyanobacteria, zooplankton, and planktivorous fish). We observed the dynamics of the phytoplankton structure, the concentrations of microcystins and chlorophyll-a, hydrochemistry, and the status of zooplankton and fish in both types of microcosms with and without algicide for one month (from 19 July to 19 August 2021). The introduction of algicide caused changes in phytoplankton structure, a drop in cyanobacterial biomass, and a decrease in the total concentration of microcystins. Surprisingly, the contributions of the most toxic microcystins (LR form) were higher in both types of microcosms exposed to algicide than in microcosms without algicide. The inhibitory effect on the cyanobacterial biomass was most significant in complex ecosystems (containing fish), while it was only observed at the end of the exposure in simple ecosystems. Not only algicide but also phytoplankton consumed by fish and zooplankton, as well as nutrient excretory activity by both consumers, seem to have impact on cyanobacterial biomass. This study found that the using chemical substances similar to macrophyte metabolites can help regulate HABs and cyanotoxins. However, the results differ depending on ecosystem type.

## 1. Introduction

Cyanobacteria and microalgae, as the principal producers of organic matter in aquatic ecosystems, are the important links in the food chains of diverse consumers, from the most basic to the highest levels. It is known that during summer when there is a nitrogen deficit in a water body, cyanobacteria are able to fix atmospheric nitrogen and thereby provide a nitrogen-rich trophic resource, enhancing the ecosystem’s production [1]. Because of anthropogenic impacts, eutrophication, and global warming, harmful algal blooms (HABs) are currently on the rise, posing a severe threat to the safety of freshwater ecosystems and drinking water. Cyanobacteria are therefore regarded as harmful components of aquatic ecosystems. Many species of cyanobacteria from genera such as *Microcystis*, *Dolichospermum* (*Anabaena*), *Planktothrix*, *Raphidiopsis*, and *Aphanizomenon* (up to 70% in fresh waters) have genes encoding toxin synthesis and can produce and release toxins into water [2,3]. Secondary metabolites (cyanotoxins) released by them during the mass development of cyanobacteria, as well as during bloom senescence and cell lysis, negatively affect the survival of various aquatic organisms, disrupting food–web links and reducing the quality of food for grazers, i.e., herbivorous zooplankton [4,5]. One of the top priorities for eutrophic water bodies is reducing the production of cyanobacteria and cyanotoxicity.

What is known in ecology about why cyanotoxins are formed? One of the hypotheses favors the signal function, i.e., the production of cyanobacterial toxins as a type of “signal” molecule by which bacterial populations can regulate their own growth depending on environmental conditions [6]. It is also suggested that cyanotoxins are produced by cyanobacteria as a form of “chemical tool” for suppressing other phytoplankton species during competitive relationships [7,8]. However, the true mechanism of metabolite formation has not been established and is still under investigation. Allelopathy between cyanobacteria and aquatic plants (macrophytes) is well recognized in nature; it can manifest itself when variables (light, nutrients) are limited, and even tolerant phytoplankton species might be vulnerable to the action of metabolites from other plants [9].

Aquatic macrophytes have a well-known inhibitory influence on harmful cyanobacterial blooms. Macrophytes can limit cyanobacterial growth through a variety of mechanisms, including the release of active substances (allelochemicals) having algicidal action [10]. Many macrophyte species actively produce these substances that inhibit the growth of cyanobacteria [11,12,13,14,15,16]. According to a review [17], 67 species of aquatic macrophytes from the genera *Cabomba*, *Myriophyllum*, *Ceratophyllum*, *Elodea*, *Nuphar*, *Stratiotes*, and the family Characeae are able to exhibit allelopathic activity against cyanobacteria. The use of aquatic macrophytes to prevent HABs is an effective method, but this approach has its own difficulties, including the need for further removal of macrophyte biomass and control of allochthonous nutrients [10]. Therefore, in order to combat cyanobacteria, it looks promising to develop approaches based on the use of substances similar in composition to allelochemicals of aquatic macrophytes to inhibit cyanobacterial bloom.

A number of studies conducted over the past several decades have demonstrated the efficiency of macrophyte allelochemicals against cyanobacteria [18,19,20,21,22,23,24], providing adequate rationale for the use of nature-like technology to combat HABs. Allelochemicals from *Myriophyllum spicatum*, for example catechin, eugeniin, and ellagic, gallic, and pyrogallic acids, inhibit the cyanobacterium *Microcystis aeruginosa* [23,24]. Following the identification of the polyphenols, artificial culture solutions have been created that contributed 50% of the allelopathic effects of *M. spicatum* on *M. aeruginosa* compared to natural culture solutions [23]. Many studies [11,25,26,27,28,29,30,31,32,33,34] have demonstrated that macrophyte extracts, which are natural compounds made from aquatic macrophytes and their synthetic equivalents, may inhibit the growth of various species of microalgae and some cyanobacteria (*M. aeruginosa*) in eutrophic water bodies. These allelochemicals are more effective than traditional algicides because of their low toxicity and excellent environmental friendliness, which offers up a new perspective on the control of harmful algae [35]. At the same time, the establishment of nature-like allelopathic technology for managing aquatic ecosystems continues relatively slowly [35,36,37,38,39]; created using synthetic equivalents of macrophyte metabolites, a new generation of 4-component algicide against cyanobacteria was recently developed [40]. Its development was based on the world literature on the allelopathy of macrophytes to cyanobacteria and the results of experimental studies [33,41].

The primary goal of the study was to determine the possible algicide effect of aquatic macrophyte metabolites (a 4-component algicide) on phytoplankton structure, cyanobacteria development, and cyanotoxin production. There was no extraction of allelochemicals from macrophytes, but pure synthetic equivalents of natural metabolites (heptanoic, octanoic, tetradecanoic, and gallic acids) were used as substitutes for macrophyte original metabolites. The allelochemicals used by us (specifically gallic acid, tetradecanoic, octanoic, and heptanoic acids) are synthesized by many macrophytes and have an allelopathic effect on cyanobacteria, as demonstrated in previous research and numerous literature sources summarized in the review paper [33].

We expected that the intensity of the cyanobacterial bloom and production of cyanotoxins would be affected by the presence of algicide and the complexity of the structure of aquatic animal communities. We predicted that algicides would limit cyanobacterial development through allelopathy, i.e., decreasing their biomass, functional activity (chlorophyll-a), and microcystin production, and therefore overall reducing the adverse influence of HABs. At the same time, we would like to clarify whether the algicide used to control cyanobacterial blooms has a negative influence on other phytoplankton and food-web participants (zooplankton and fish) in the aquatic ecosystem. Another critical challenge was determining how the algicide’s inhibitory effect will express itself in a simple ecosystem with only zooplankton and in a complex ecosystem with the presence of zooplankton and planktivorous fish. In the first scenario, preliminary data [42] suggested that planktonic crustaceans would contribute significantly to the growth of cyanobacteria by releasing nutrients (mostly dissolved orthophosphates). At the same time, fish, on average, release nutrients with less intensity than zooplankton [42], although they may limit zooplankton activity due to predation (top-down effect). Our findings could help researchers better understand how allelopathy and plant-derived active compounds with cyanobacterial inhibitory properties can be employed to improve environmental water quality.

## 2. Results

### 2.1. Phytoplankton

Altogether, 60 species of phytoplankton were identified in I, II, and III, and 66 species in IV (Table S1). The start conditions in the treatments are shown in Table 1. The species composition was quite similar for cyanobacteria (11 species were recorded in each treatment). Chlorophytes were the main contribution to the total species richness; 40 species were registered in this group. Notable differences in the number of species were found between treatments for Chlorophyta (I: 25, II: 28, III: 22, and IV: 31 species; Table S1).

Table 2 presents the succession of dominant species, which accounted for from 65 to 98% of the total biomass of the community. Several species (*Aphanizomenon flos-aquae*, *Microcystis aeruginosa, Aulacoseira granulate, Dolichospermum spiralis,* and *Cryptomonas* spp.) were identified as dominant species in terms of biomass for all treatments. At the same time, phytoplankton communities in III and IV were richer in species than in I and II due to the development of some species of Charophyta.

**Figure 1:**
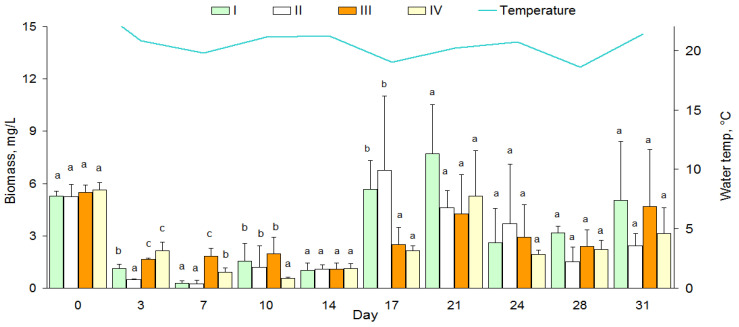
Total biomass (mg/L) of phytoplankton (left y-axis) and water temperature (°C at 9:00 a.m., right y-axis) in microcosms during the exposure period. Treatment conditions are shown in Table 1. Different letters between pairs show significant differences (*p* < 0.05) for each date (day), but the same letters (a, b, c) do not.

Total phytoplankton biomass (Figure 1) and cyanobacterial biomass (Table 3) differed significantly between dates (one-way ANOVA, all *p* < 0.01); however, there were no significant differences in the biomass of these taxa between treatments (Friedman test: *χ*^2^ = 1.92; *p* = 0.59 and *χ*^2^ = 1.56; *p* = 0.68). The total biomass of phytoplankton showed strong relationships between treatments (Spearman: *Rs* = 0.67–0.92, *p* = 0.0002–0.03). The decrease in phytoplankton biomass observed in all microcosms at the beginning of the experiment was due in part to worsening weather conditions, such as high cloudiness, wind, rain, and a decrease in water temperature (Figure 1). However, no significant correlations between water temperature and phytoplankton biomass were found (*R_s_* = 0.49–0.56, *p* = 0.09–0.15).

**Figure 2:**
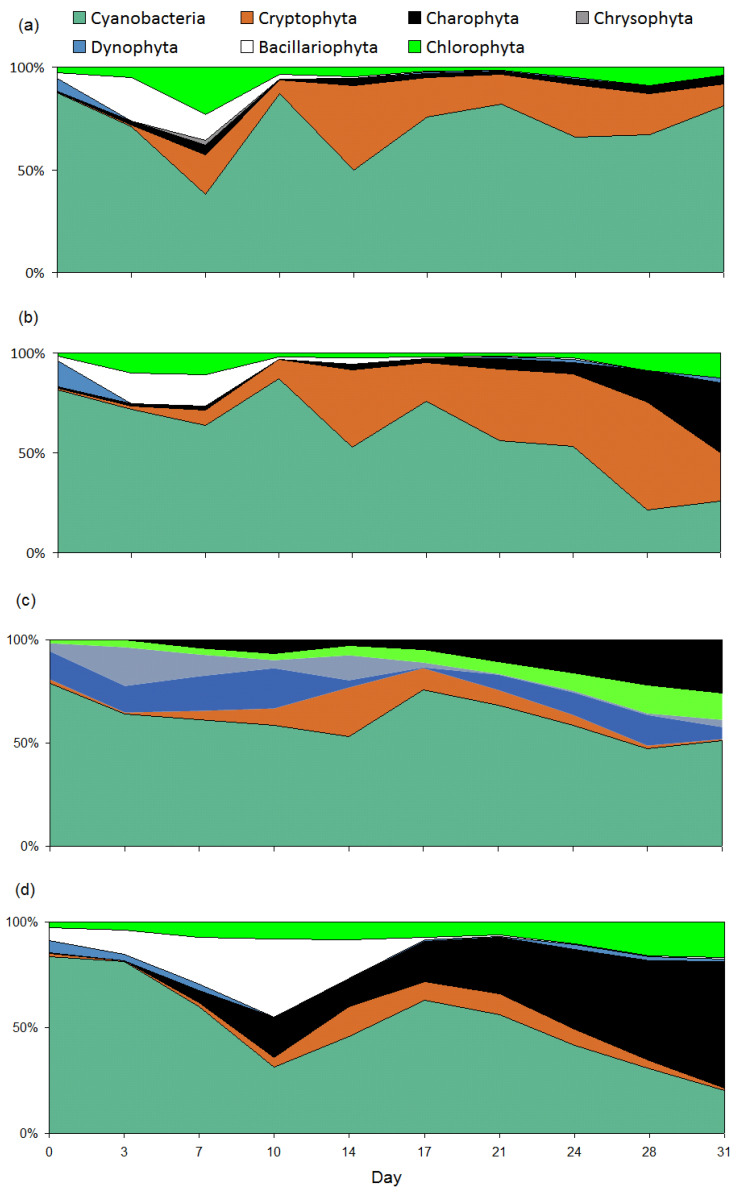
Percentage contribution (%) of the main taxonomic groups to the total phytoplankton biomass in four types of microcosms (I, II, III, and IV) during exposure. The abscissa shows the day of observation. (**a**) I; (**b**) II; (**c**) III; (**d**) IV.

Cyanobacteria and cryptophytes predominated in I, II, and III (according to biomass) (see Figure 2, Table 2). In IV, in addition to cyanobacteria, a significant contribution to the total biomass was made by charophytes (Desmidiales). Significant differences were also found (Friedman: *χ*^2^ = 9.96; *p* = 0.02) in the biomass of cryptophytes between I and II (*p* = 0.04), I and IV (*p* = 0.007), and II and IV (*p* = 0.005) as well as charophytes (*χ*^2^ = 15.75, *p* = 0.001) between I and III (*p* = 0.005), I and IV (*p* = 0.03), II and III (*p* = 0.007), and II and IV (*p* = 0.038).

The dynamics of the average biomass of cyanobacteria were characterized by three increased biomass values (peaks): at the beginning, mid-exposure, and at the end of exposure; and two falls (Figure 1). In addition, biomass values varied significantly between simple and complex types of communities. The second biomass peak of cyanobacteria (mid-exposure, 5–9 August) did not differ significantly (*p* > 0.05) between I and II, as well as III and IV (Table 3). However, these maximum biomass values of cyanobacteria were about two times lower in III and IV than in I and II (Table 3). We found significant differences in the biomass of cyanobacteria between I and II (4.1 vs. 0.60 mg/L) and between III and IV (2.78 vs. 0.83 mg/L) at the end of exposure.

### 2.2. Cyanotoxins

**Figure 3:**
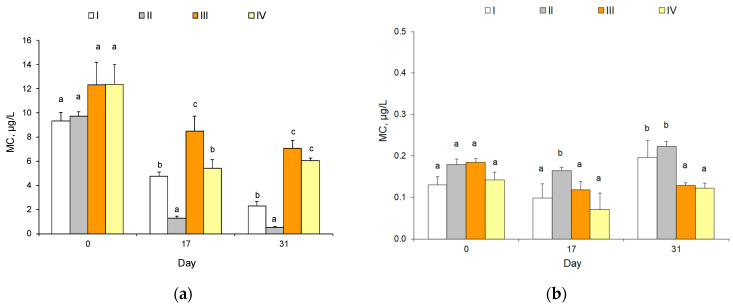
Mean concentrations of microcystins (MC, µg/L) in four treatments (I, II, III, and IV) at the beginning (start, 19 July/day 0), the middle (mid-exposure, 5 August/day 17), and the end of exposure (end, 19 August/day 31). Mean value ± 95% confidence intervals, n = 3. (**a**) Concentrations of intracellular microcystins accumulated in the biomass of cyanobacteria; (**b**) concentrations of extracellular microcystins found in water. Different letters show significant differences (*p* < 0.05) between pairs on date.

Figure 3 outlines the total concentrations of microcystins (MCs) in microcosms. At the beginning of exposure, the concentrations of intracellular MCs contained in the biomass of cyanobacteria were approximately the same in all treatments and varied from 9.3 to 12.4 µg/L. The dissolved MC concentrations in water ranged from 0.09 to 0.24 µg/L. During exposure, the total content of intracellular MCs decreased in all treatments, but this was most obvious in II (Figure 3a, Table 4). In water, the total content of MCs remains rather constant (Figure 3b). Total MC concentrations differed significantly between treatments (χ^2^ = 9, *p* = 0.01) and between dates (excluding day 0) across all treatments (χ^2^ = 7.42, *p* = 0.024), statistically confirming the mid-exposure period (*p* = 0.03) and end of exposure (*p* = 0.03).

We found that the percentage proportion of the most toxic form, such as MC-LR, was 69–76% in all treatments at the beginning of the exposure and decreased toward the end (Table 4, see Table S2 for statistical differences in forms between treatments). The contribution of MC-LR to the total concentration of MCs was lower in microcosms without algicide (I and III) compared to microcosms with algicide. In IV, the relative contribution of MC-LR remained at a high level (21–28%), reaching 1.25 µg/L.

### 2.3. Chlorophyll-a

**Figure 4:**
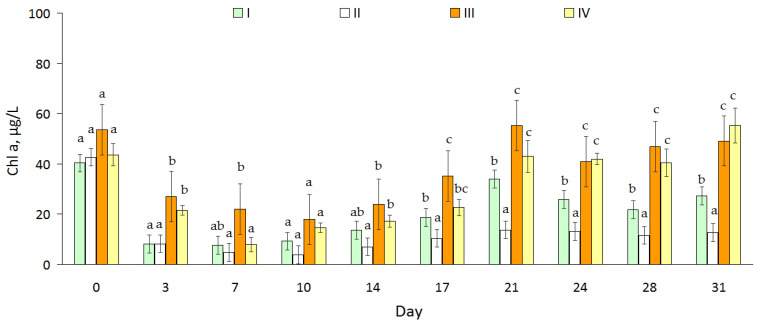
Concentration of chlorophyll-a (μg/L) in water of four treatments (I, II, III, IV), mean value ± 95% confidence intervals, n = 3. The different letters show significant differences (*p* < 0.05) between pairs for each date (*p* < 0.05).

Concentrations of chlorophyll-a (Chl-a) were significantly higher in III and IV compared to I and II (Figure 4, all *p* < 0.05). Also, the concentration of Chl-a in II was significantly lower than in I. There were significant differences between I and III (Friedman: χ^2^ = 10.8, *p* = 0.001). In addition, the concentration of Chl-a in II was significantly lower than in I (χ^2^ = 13.3, *p* = 0.0003). The concentration of Chl-a in the treatments without algicide (I and III) correlated with the total phytoplankton biomass (according to Spearman: R_s_ = 0.88 (*p* = 0.008) and 0.71 (*p* = 0.02), respectively), whereas in the treatments with algicide there were no such relationships (R_s_ = 0.54, *p* = 0.11 (II) and 0.52, *p* = 0.13 (IV)).

### 2.4. Salt Content, Nutrients, and Physical Parameters of Water

Table 5 shows the chemical and physical parameters of the water during whole period of exposure (n = 10). There were no significant differences in the average values of chemical variables between treatments. Also, the dynamics of the concentrations of basic cations and anions and also total nitrogen in the water were similar for all treatments: significant differences were not found on each date (all *p* > 0.05). In IV, a slight increase in Ca^2+^ (Friedman test: χ^2^ = 14.22, *p* = 0.0002) and HCO_3_^−^ (χ^2^ = 9, *p* = 0.003) compared to III was noted on some dates (Figure S2). In addition, the pH in IV was lower than in III (χ^2^ = 24.14, *p* < 0.0001), and the same trend was noted for the concentration of dissolved oxygen (χ^2^ = 7.14, *p* = 0.009, Figure S2). Microcosms III and IV were distinguished by a higher average concentration of total phosphorus than microcosms I and II (Table 5). Significant differences in total phosphorus were established between I and III (χ^2^ = 7.12, *p* = 0.009), as well as between II and IV (χ^2^ = 12.25, *p* = 0.0005, Figure S2).

### 2.5. Zooplankton

**Figure 5:**
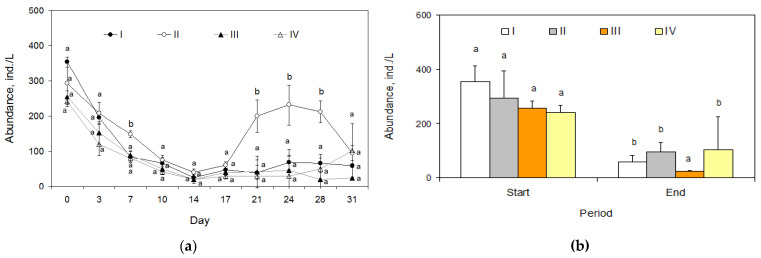
Abundance (individuals per L, ind./L) of zooplankton: (**a**) dynamics of abundance of planktonic crustaceans Cladocera in treatments I, II, III, and IV (mean ± 95% confidence intervals, n = 3); (**b**) the mean total abundance of zooplankton (±95% confidence intervals, n = 3) of zooplankton (ind./L) in four treatments (I, II, III, and IV) at the beginning (Start) and the end of exposure (End). The significant differences (*p* < 0.05) are shown by different letters.

Copepoda, Cladocera, and Rotifera were the three major taxonomic groups of zooplankton. The dynamics of abundance for two groups of them (Copepoda and Rotifera) did not differ significantly between treatments (χ^2^ = 0.31, *p* = 0.6, and χ^2^ = 1.29, *p* = 0.27, respectively); algicide may not have affected the abundance of these taxa. The dynamics of Cladocera abundance were characterized by significant differences between I and II (χ^2^ = 13.3, *p* = 0.0003) and an increase in the second half of the exposition period, with II showing 1.5-fold larger abundance than I (Figure 5a). This was due to a change in the dominant species (from *D. longispina* to *Ceriodaphnia quadrangula*). There were no differences in abundance between III and IV (all *p* > 0.05); However, the abundance of Cladocera was lower in these treatments than in I and II due to active consumption of planktonic crustaceans by fish. Significant differences between the start and end abundance (a decrease) of these crustaceans were found in treatments I (*t*-test; *p* = 0.0001), II (*p* = 0.027), and III (*p* = 0.0006), while in IV this difference was also lower by the end of exposition but not significantly (*p* = 0.125, Figure 5b). Cladoceran abundance in III was significantly lower (3 times) than in I at the end of the exposure (*t*-test; *p* = 0.0001), but similar differences between II and IV were not significant (*t*-test; *p* = 0.902). The end abundance of Cladocera did not differ between I and II (*t*-test; *p* = 0.078), III, and IV (*p* = 0.258). In III and IV, fish also controlled the abundance of copepod crustaceans, keeping them at a minimum. At the same time, the abundance of Rotifera grew significantly, reaching 688 ind./L in III and 494 ind./L in IV at the end of the experiment, but their biomass contributions were insignificant due to small body sizes.

### 2.6. Fish

**Figure 6:**
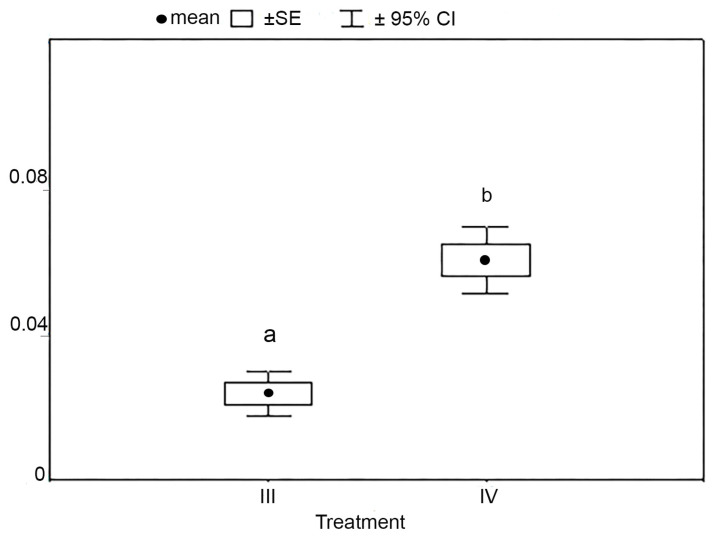
Mass growth rate (g/month) of medaka (fish) under exposure conditions III and IV (n = 45). SE is the standard error, and 95% CI is a 95% confidence interval. Significant differences (*p* < 0.05) are shown by different letters.

There were no significant differences in the fatness of fish at the beginning and end of the experiment (χ^2^ = 2.87, *p* = 0.72, and χ^2^ = 2.93, *p* = 0.71). Significant differences in the final mass of fish were found between III and IV (χ^2^ = 11.79, *p* = 0.038), and no significant differences between III and IV were found in the initial mass of fish (χ^2^ = 4.24, *p* = 0.52). It was found that the rate of fish mass growth rate of fish during the period of the exposure differed between III (0.023 g/month) and IV (0.06 g/month; *t*-test, *p* = 0.009, Figure 6). By the end of the experiment, the fish in IV had a greater mass than the fish in III due to a faster increase in mass by 64.9%. A similar trend was noted for the rate of length growth.

## 3. Discussion

The results obtained showed that the metabolites of aquatic macrophytes in the composition of the 4-component algicide [40] used in the experiment altered phytoplankton structure, cyanobacterial biomass, and cyanotoxins concentration. The structure of phytoplankton community differed depending on the type of ecosystem (simple and complex). The community became more diverse with the addition of an algicide due to the increase in the number of species from the order Chlorophyta. Other studies have found that when plant-derived active compounds prevented cyanobacteria and microalgae blooms, phytoplankton diversity and species homogeneity increased [17].

According to the study, food-web interactions first affect the composition of dominant species, and then species interactions cause a change in the structure of communities. Phytoplankton succession was expressed in the change of dominant species from cyanobacteria to cryptophytes, and then to charophytes. In the absence of fish (a simple ecosystem), the biomass of cryptophytes increased rapidly, and the second peak of phytoplankton biomass was clearly apparent. In the presence of fish (III and IV) a very different succession was observed in the presence of fish (III and IV), with the charophytes, *Mougeotia* sp. and *Staurastrum* sp., developing successfully and the second peak of cyanobacterial biomass being less evident than in simple ecosystems. Perhaps the medaka could use cryptophytes and other microalgae as food, giving charophytes an advantage in development. Mixotrophic cryptomonads, in particular, are recognized to be optimal food for planktivorous consumers [43]. Various species of the genus *Oryzias* (medaka) exhibit strategies of herbivores (eating phytoplankton [44]), omnivores prone to herbivory [45], or consumers of insects and microalgae [46].

Phosphates and nitrates (mineral forms of nutrients) were present at low concentrations in all treatments; they were actively consumed by phytoplankton for biomass growth. The presence of fish (III and IV) resulted in a greater total phosphorus level, indicating better trophic conditions. Fish excrete a lot of organic phosphorus, and bacteria and microalgae can produce extracellular phosphatase for the hydrolysis of organic phosphorus, compensating for a lack of available mineral phosphorus in the environment [47]. This will benefit phytoplankton, particularly for nitrogen-fixing cyanobacteria under nitrogen-deficient conditions.

We expected that algicide would cause a drop in the Chl-a concentration due to changes in the structure of phytoplankton and a decrease in the biomass of cyanobacteria. This proposition has been proven in simple ecosystems where there were no fish. The level of Chl-a was significantly (almost two times) greater in complex ecosystems (containing fish) than in simple ecosystems. The productivity of water bodies is determined by the inflow of nutrients from the watershed as well as nutrient regeneration within the ecosystem. Although planktonic crustaceans had a higher intensity (per unit of mass) of phosphorus regeneration than fish [42], given that the biomass of fish was much higher than that of planktonic crustaceans, such a high level of productivity in the presence of fish is quite understandable.

Cyanobacteria genera that dominated all treatments, such as *Microcystis, Dolichospermum* (formerly *Anabaena*), and *Aphanizomenon,* are known to produce cyanotoxins in freshwater [46], which might alter food–web interactions and reduce food quality for herbivorous zooplankton. Our assumption that the intensity of the cyanobacterial bloom will be determined by the presence of algicide and the complexity of the structural organization of aquatic animal communities was confirmed. The concentration of cyanotoxins was also related to the presence of algicide and the type of microcosm. The degree of cyanotoxin release varies between cyanobacteria species and even between strains of the same species in various environmental conditions [48,49,50]. However, the quantity of toxic microcystins (MC-LR) accumulated on bacterial cells and dissolved in water remained at a level of 20–30% in the presence of algicide, in contrast to treatments without it (where MC-LR decreased to 5–10%).

The presence of algicide obviously enhances cell lysis and the subsequent release of microcystins (from cells into water). The large contribution of toxic MC-LR during algicide action may be caused by a decrease in cyanobacterial abundance, indicating a signal mechanism for the production of cyanobacterial toxins that aid cyanobacteria in regulating their own growth [6]. Also, this could be a cyanobacterial response to allelochemicals (the imaginary presence of competitors) and may indicate the inclusion of an allelopathic suppression mechanism from the side of cyanobacteria against other plant organisms [7]. Fortunately, in this study, the most toxic variants (MC-LR) were mostly below the water guideline levels (i.e., 1 µg/L) [51]. Future research should consider how cyanotoxins might accumulate along food chains, despite the decrease in their abundance in water due to grazing by zooplankton and fish [52,53,54,55,56]. The medaka fish (*O. latipes*) used in our experiment accumulated microcystins in the presence of cyanobacteria as well [56].

Interestingly, macrophyte metabolites (algicide) that inhibit cyanobacteria had no negative effects on other phytoplankton or other participants in the food web, such as zooplankton and fish. The use of algicide had a positive effect on the abundance of cladocerans and the mass growth of fish. This beneficial effect appears to have been mediated by a change in phytoplankton composition, specifically an increase in the proportion of charophytes (desmids) and cryptophytes, which are better food for consumers than cyanobacteria.

The presence of toxic cyanobacteria often leads to a change in zooplankton towards the predominance of small species [57,58,59]. Changes in the structure of Cladocera were indeed significant due to the change in species dominance from large *Daphnia* to small *Ceriodaphnia*. Unlike the generalist *Daphnia*, the smaller *Ceriodaphnia* is able to feed more selectively and thus develop stronger resistance to cyanotoxins [59,60]. Analysis of carbon and nitrogen isotopes showed that *Ceriodaphnia* did not graze directly on colonial or filamentous cyanobacteria, preferring to feed small algae and bacteria (particles < 20–100 µm [60]). In fact, other cladoceran *Bosmina* and calanoid copepods can ingest some cyanobacteria: *Aphanizomenon*, *Microcystis, Dolichospermum* and *Merismopedia tenuissima* [57,61,62,63]. Direct grazing by zooplankton may be an important factor contributing to the decrease in the biomass of cyanobacteria [62,63]. However, zooplankton consumption of cyanobacteria reduces their biomass to a lesser extent than fish, which can trigger cascade effects and hence limit cyanobacteria and other phytoplankton abundance.

One of the most active areas of practical aquatic ecology and water management is the suppression of cyanobacteria and microalgae by active compounds (allelochemicals) of macrophyte origin or their synthetic equivalents [17,35,36,37]. Synthetic active compounds with good biological safety, similar to macrophyte metabolites, and pronounced specific action against cyanobacteria make them promising for use in HAB suppression. The findings from our study support the use of an algicide. Despite all the positive aspects, however, we would like to emphasize that in complex aquatic ecosystems with the presence of fish, internal nutrient load and trophic interactions are extremely important and can regulate the level of development of cyanobacteria and other groups of phytoplankton, nullifying and negating the effectiveness of algicide. This point is important to take into account when planning activities to combat cyanobacteria using algicides. Furthermore, the tendency we noted towards an increase in toxic forms of microcystins in the environment under the action of the applied algicide still leaves the mechanism of their production by cyanobacteria and their appearance in the environment unexplained. In this regard, further research on the inhibition of cyanobacteria using active substances of plant origin or their equivalents should take into account the ability of some types of cyanobacteria to release toxic cyanotoxins. They should determine those threshold concentrations of metabolites or active substances that will not lead to an increase in the cyanotoxicity of the environment above an acceptable level.

## 4. Materials and Methods

### 4.1. Experiment Design

The study was carried out on the basis of the experimental work station of the Papanin Institute for Biology of Inland Waters of the Russian Academy of Sciences (IBIW RAS, Borok, Russia, 58°02′ N; 38°14′ E). Microcosms were created in plastic fish trays (100 × 100 × 40 cm) and placed in a water pool to avoid high diurnal fluctuations in water temperature. The trays were filled with natural water (300 L) from a tributary of the Volga River (Rybinsk Reservoir) filtered through a sieve with a mesh size of 63 μm. The trays were isolated with mesh from animals from the air and from the pool (Figure S1).

We used four treatments with three identical replicates and various ecosystems with and without algicide (Table 1). All treatments had comparable phytoplankton and cyanobacteria biomasses and zooplankton abundances. Natural phytoplankton, bacteria, and parts of zooplankton were entered into treatments along with added water.

The cyanobacteria *A. flos-aquae* and *Microcystis aeruginosa* were taken from a shallow artificial pool with a high development of these species of cyanobacteria to introduce in microcosms. We concentrated 200 L of water containing cyanobacteria to a volume of 1 L using a plankton net with a mesh size of 63 μm and then added it to each tray in similar concentrations (Table 1).

Zooplankton was caught in an artificial pond with the same river water, concentrated in a separate container, and then added in approximately equal amounts to each tray (Table 1). The cladocerans (*Daphnia longispina*) dominated the community and accounted for 40% of the total abundance of zooplankton in the introduced zooplankton.

Adult fish (medaka) from the cultural strain Hd-rR, ten months old, were obtained from the Laboratory of Physiology and Toxicology of Aquatic Animals, IBIW RAS. A selection of 15 individuals of fish were placed in each tray. Their body length varied from 27.1 to 28.2 mm, and their average mass varied from 0.416 to 0.467 g. In each tray (300 L) of treatments III and IV, we added fish (15 individuals per tray).

In each tray (300 L) of treatments II and IV, we added 15 mL of a four-component algicide. The concentration of this algicide in each microcosm was 0.05 mL/L. The total concentration of metabolites in water was approximately 14 mg/L. The algicide and fish were introduced on 19 July 2021, after the first collection of phytoplankton and zooplankton (Table 1) and chemical samples.

### 4.2. Algicide

To generate an algicide, purified chemicals, such as heptanoic acid, octanoic acid, tetradecanoic acid, and gallic acid, produced by Acros Organics BVBA (https://www.acros.com/ accessed on 1 January 2020), were purchased. These chemical compounds are macrophyte metabolites [35]. The algicide was prepared from four organic acids and ethanol in the following concentrations (25% of active compound units): gallic acid C_7_H_6_O_5_ (70 g/L); tetradecanoic acid C_14_H_28_O_2_ (70 g/L); heptanoic acid C_7_H_14_O_2_ (70 g/L); octanoic acid C_8_H_16_O_2_ (70 g/L); and ethyl alcohol (1 L) [40]. The suggestion that allelochemicals could have a synergistic effect led to the use of a 4-component composite. The 1:1:1:1 mass ratio of heptanoic, octanoic, tetradecanoic, and gallic acids in the composition of the algicide was chosen based on the task of achieving maximum dissolution in ethanol as a matrix containing active components and eliminating the advantage of any component. Ethyl alcohol was the most suitable solvent, allowing all of the used acids to be dissolved in a minimum volume. The effect of this algicide in water can be achieved at a concentration of 0.01 mg/L [64].

### 4.3. Samples Collection and Laboratory Procedures

Phytoplankton, zooplankton, and water samples for chlorophyll-a pigment (Chl-a) and chemical analyses were sampled on 19 July before algicide and fish release and then every three days during exposure (from 22 July to 19 August 2021). During this sampling, surface water temperature and pH were measured using a thermometer and a multi-parameter liquid analyzer, Ecotest 2000.

Phytoplankton samples (volume 0.5 L) were collected in the morning in the surface layer of water from each tray using an original pipette-type sampler. A fixative composed of two solutions was used to preserve the sample: Lugol’s solution (10 g potassium iodine + 50 mL H_2_O + 5 g iodine) and a solution of 10 mL glacial acetic acid and 80 mL 4% formalin. This fixative (1.5 mL) was added to a 0.5 L sample. To analyze the composition of algae and planktonic bacteria, the samples were settled for ten days and siphoned to a 10 mL volume. Species identification and individual cell counting were carried out in a Nageotte chamber with a volume of 0.02 mL using an optical microscope, the Bioptic B-200 (Biomed, St. Petersburg, Russia), at 600 magnifications. Species biomass was calculated using species-specific geometric formulas [65]. To determine the concentration of Chl a, 0.2-L water samples were filtered through a cellulose nitrate filter (3 μm of pore diameter, Vladisart, Russia) with a substrate of glass powder and calcium carbonate. Filters with seston deposited were stored at a temperature of −20 °C.

Samples of microcystins were collected three times: at the beginning, mid-exposure, and at the end of the experiment. We separated water samples of microcystins into water and biomass by filtration using a Vladipor 0.45 μm membrane filter. After filtration, the water and filters were stored at a temperature of −20 °C.

Water for zooplankton analysis was taken at four points in the microcosm with a 0.5 L sampler of the original design. The total sample from each microcosm was 2 L. Zooplankton samples were conserved with 70% ethanol. The zooplankton species identification and counting of abundance were carried out in Bogorov’s chamber under a stereoscopic microscope (Leica MZ 9.5).

Fish were counted and weighed before the experiment and at the end of the exposure (in 30 days). Their wet biomass was calculated as a minus between the weighing of a small cuvette filled with water with and without fish. The mass was determined on an analytical balance (AND HR-150AZ, Tokio, Japan) with an accuracy of 0.1 mg. The individuals were weighed in a cuvette with 2 mL of pure water. Each fish was carefully caught with a net and placed on filter paper to remove water, after which it was placed in a cuvette and weighed. The weight of the fish was determined by the difference between the cuvette with and without the fish. The length of the fish was determined using a ruler placed on a transparent cuvette with an accuracy of 0.5 mm. The fatness coefficient (Fulton’s condition factor, *K*) was calculated using the formula: *K* = 100 × *m*/*l*^3^, where *m* is the mass of the fish in grams and *l* is body length in cm estimated from the end of the snout to the end of the scaly cover.

### 4.4. Chemical Methods and Protocols

Cyanotoxins were detected using the high-performance liquid chromatography-high-resolution mass-spectrometry (HPLC-HRMS) method. Mass-spectrometric analysis was carried out under electrospray ionization in the positive ion detection mode. The identification of target compounds was based on the accurate mass measurement of [M + H] or [M + 2H]^2+^ ions (resolution of 30,000, accuracy within 5 ppm), the collected fragmentation spectrum of the ions, and the retention times. The limit of detection was 2 ng/L.

Sample preparation included extraction of the dissolved extracellular cyanotoxins from filtered water using Oasis HLB SPE cartridges (Waters) and extraction of intracellular toxins fraction from biomass collected on filters with 1 mL of 75% methanol in an ultrasonic bath, according to [66]. We use the LC-20 Prominence HPLC system (Shimadzu, Japan) coupled with the LTQ Orbitrap XL Hybrid Ion Trap and Orbitrap Mass Spectrometer (Thermo Fisher Scientific, San Jose, CA, USA) to analyze extracts. Toxins were separated by gradient elution (0.2 mL/min) with a combination of water and acetonitrile containing 0.05% formic acid on a Thermo Hypersil Gold RP C18 column (100 mm × 3 mm, 3 μm) with a Hypersil Gold dropin guard column (Thermo Fisher Scientific).

All chemicals used for analytical procedures were the analytical grades: acetonitrile (HPLC-grade) and methanol (LiChrosolv hypergrade for LC-MS) from Merck (Darmstadt, Germany) and formic acid (98–100%) from Fluka Chemika (Buchs, Switzerland). High-quality water (18.2 MΩ/cm) was obtained by the water purification system Millipore Direct-Q (Bedford, MA, USA). We purchased MC standards from Sigma Aldrich, St. Louis, MO, USA (MC-LR, MC-RR, and MC-YR) and Enzo Life Sciences, New York, NY, USA (MC-LY, MC-LA, MC-LW, MC-LF, [D-Asp^3^] MC-LR, and [D-Asp^3^] MC-RR).

The phytoplankton Chl-a was determined on a METASH V-5000 spectrophotometer (Shanghai, China) using the method in [67], the pigments were extracted with 90% acetone before analysis. The capillary electrophoresis on an electrophoresis equipment (Kapel-105, OOO Lumex, Russia) was used to evaluate the concentrations of ions K^+^, Na^+^, Mg^2+^, Ca^2+^, Cl^−^, and SO_4_^2−^ in water [68,69]. Titrimetric analysis was used to evaluate the concentrations of bicarbonates and total nitrogen content in water [70,71]. The total phosphorus was detected in unfiltered water after mineralization of the sample to orthophosphates and subsequent oxidation with persulfate [72]. The dissolved oxygen concentration in water was measured by the iodometric method [73].

### 4.5. Statistics

We calculated the mean of values, standard deviation, and coefficient of variation for the subsequent statistical analyses. All data were tested for normality with the Kolmogorov–Smirnov test and for homogeneity of variances with Levene’s test. For comparison of normally distributed data (abundance and biomass of zooplankton groups; abundance of phytoplankton between dates and sites), ANOVA (analysis of variance, F-test), followed by Tukey’s pairwise comparison, was used. For nonparametric data, differences between the variables were evaluated by a non-parametric Χ^2^-test (Friedman test), followed by Wilcoxon test and tests with Bonferroni correction for pairwise comparisons. Spearman rank correlation (*R_s_*) was applied to find relationships between variables and environmental factors. Significant differences between the groups of variables were accepted at *p* < 0.05.

## Figures and Tables

**Table 1:** Scheme of experiment and start conditions (19 July) of zooplankton and fish abundance (individuals/m^2^), mean concentration of cyanobacteria, other phytoplankton, and concentration of algicide. The data are presented as a mean value for three replicates with a standard deviation. No significant differences in mean values between treatments were observed for each variable assessed.

	Treatment			
	I	II	III	IV
Phytoplankton, mg/L	1.04 ± 0.42	0.93 ± 0.43	1.04 ± 0.41	0.84 ± 0.34
Cyanobacteria, mg/L	4.29 ± 0.21	4.25 ± 0.18	4.34 ± 0.23	4.69 ± 0.34
Zooplankton, ind./L	616 ± 113	613 ± 156	636 ± 139	659 ± 156
Fish ^1^	No	No	15	15
Algicide ^2^, mg/L	No	14	No	14

^1^ medaka *Oryzias latipes*; ^2^ algicide is mix of heptanoic, octanoic, tetradecanoic, and gallic acids (1:1:1:1).

**Table 2:** Succession of key species of phytoplankton contributing 60–98% to the total community biomass in four treatments (I, II, III, IV) during the experimental exposition.

Date (Day)	I	II	III	IV
19–26 July (7)	*Aphanizomenon flos-aquae* *Microcystis aeruginosa* *Aulacoseira granulata*			
29 July (10)	*A. flos-aquae*	*A. flos-aquae* *Cryptomonas* spp.	*A. flos-aquae* *M. aeruginosa*	*M. aeruginosa Mougeotia* sp. *A. granulata*
2 August (14)	*Cryptomonas* spp. *Dolichospermum spiralis* *A. flos-aquae*		*D. spiralis* *A. flos-aquae* *Cryptomonas* spp.	*D. spiralis* *A. flos-aquae* *M. aeruginosa*
5 August (17)	*A. flos-aquae* *Cryptomonas* spp. *D. spiralis*	*A. flos-aquae* *Cryptomonas* spp.	*A. flos-aquae* *Cryptomonas* spp. *D. spiralis*	*A. flos-aquae* *D. spiralis*
9 August (21)	*A. flos-aquae*	*A. flos-aquae* *Cryptomonas* ssp.	*A. flos-aquae*	*A. flos-aquae* *M. aeruginosa* *D. spiralis*
12 August (24)	*A. flos-aquae* *Cryptomonas* spp. *D. spiralis*	*Cryptomonas* spp.	*D. spiralis* *A. flos-aquae* *Staurastrum* sp.	*D. spiralis* *Mougeotia* sp. *Staurastrum* sp.
16 August (28)	*A. flos-aquae* *Cryptomonas* spp. *D. spiralis*	*Cryptomonas* ssp. *Mougeotia* sp. *A. flos-aquae*	*D. spiralis* *A. flos-aquae* *Staurastrum* sp.	*D. spiralis* *A. flos-aquae* *Staurastrum* sp. *Mougeotia* sp.
19 August (31)	*A. flos-aquae* *D. spiralis*	*Cryptomonas* spp. *Mougeotia* sp. *A. flos-aquae*	*D. spiralis*	*Mougeotia* sp. *Staurastrum* sp. *A. flos-aquae*

**Table 3:** Biomass (mg/L) of cyanobacteria at the beginning (start, 19 July/day 0), in the middle of exposure (mid-exposure, 5–9 August/days 17–21), and at the end (19 August/day 31) of the experiment in four treatments (I, II, III, IV). Mean values and 95% confidence intervals (CI) are shown; *n* = 3. Different letters show significant differences (*p* < 0.05) between treatments for each period.

Period	Value		Treatment		
		I	II	III	IV
Start	Mean 95%CI	4.29 ^a^ 0.12	4.25 ^a^ 0.10	4.27 ^a^ 0.10	4.82 ^a^ 0.08
Mid-exposure	Mean 95%CI	6.31 ^b^ 1.24	6.09 ^b^ 0.85	3.44 ^a^ 0.66	2.75 ^a^ 0.15
End	Mean 95% CI	4.10 ^c^ 1.36	0.60 ^a^ 0.17	2.78 ^b^ 1.39	0.83 ^a^ 0.20

**Table 4:** Average total concentration (with 95% confidence interval, CI) of the intracellular microcystins (MC, µg/L), n = 3, contained in the biomass of cyanobacteria, and the percentage contributions (%) of various forms of MCs to the total concentrations at the beginning (start, 19 July/day 0), the middle (mid-exposure, 5 August/day 17), and at the end (end, 19 August/day 31). “-” means no toxins were detected. Different letters show significant differences (*p* < 0.05) between pairs for each period.

MC Forms	Period of Exposure											
	Start				Mid-Exposure				End			
	I	II	III	IV	I	II	III	IV	I	II	III	IV
Mean concentration, µg/L 95% CI	9.34 ^a^ 0.72	9.76 ^a^ 0.34	12.30 ^a^ 1.90	12.38 ^a^ 1.62	4.75 ^b^ 0.35	1.28 ^a^ 0.17	8.50 ^c^ 1.25	5.43 ^b^ 0.71	2.31 ^b^ 0.34	0.51 ^a^ 0.12	7.08 ^c^ 0.67	6.04 ^c^ 0.21
% MC-LR	68.6	69.1	76.2	75.6	5.9	31.3	27.4	37.6	5.3	28.3	9.7	20.7
% [D-Asp^3^]MC-LR	3.6	3.5	3.3	3.5	6.7	7.2	5.1	6.7	9.6	7.3	6.4	8.1
% [D-Glu-OCH_3_^6^]MC-LR	1.0	-	1.1	1.2	-	-	0.3	0.4	-	-	-	-
% MC-YR	20.5	20.4	13.3	13.0	76.6	51.3	61.5	48.3	76.0	48.8	80.5	66.7
% [Dha^7^]MC-YR	5.7	5.9	5.0	5.6	10.9	10.0	5.5	6.8	9.0	12.8	3.4	4.3
% MC-LW	0.2	0.2	0.3	0.3	-	0.1	0.2	0.2	-	-	-	-
% [D-Asp^3^]MC-RR	0.1	-	0.1	0.1	-	-	-	-	-	-	-	-

**Table 5:** Mean minimum–maximum values (mg/L) of chemical parameters of water in four treatments (I, II, III, and IV) for period of exposure, n = 10. P_tot_ is total phosphorus, N_tot_ is total nitrogen, and other compounds are given chemical formulas. No significant differences in mean values between treatments were observed for each variable assessed.

Variables	I	II	III	IV
Na^+^	3.15 (2.43–3.84)	3.26 (2.43–3.86)	3.23 (2.33–3.82)	3.16 (2.23–3.80)
K^+^	2.97 (2.33–3.48)	3.00 (2.31–3.44)	3.01 (2.30–3.47)	3.07 (2.40–3.60)
Ca^2+^	23.65 (17.63–30.98)	24.46 (16.97–31.11)	22.89 (15.61–30.78)	25.25 (16.29–32.01)
Mg^2+^	6.63 (4.85–8.29)	6.80 (4.76–8.35)	6.67 (4.42–8.30)	6.70 (4.33–8.23)
Cl^−^	3.49 (2.67–4.06)	3.48 (2.45–4.22)	3.36 (2.51–3.99)	3.35 (2.44–3.93)
SO_4_^2−^	3.61 (1.64–4.84)	3.71 (1.71–5.24)	3.51 (1.31–4.67)	3.61 (1.93–4.61)
HCO_3_^−^	107.57 (80.55–138.72)	111.36 (78.31–139.53)	106.07 (72.20–133.02)	113.86 (75.78–142.79)
PO_4_^3−^	0.03 (0.01–0.08)	0.02 (0.01–0.05)	0.02 (0.01–0.03)	0.02 (0.01–0.03)
P_tot_	0.05 (0.03–0.10)	0.05 (0.03–0.07)	0.07 (0.05–0.09)	0.07 (0.04–0.08)
N_tot_	1.07 (0.96–1.16)	0.95 (0.91–0.98)	1.25 (1.17–1.36)	1.20 (1.09–1.43)
O_2_	8.63 (6.55–9.88)	8.20 (6.76–9.26)	8.34 (6.65–9.36)	7.63 (6.45–9.05)
pH	9.1 (8.8–9.3)	9.1 (8.7–9.4)	9.2 (8.8–9.4)	8.9 (8.6–9.3)

## Data Availability

The data presented in this study are available in Supplementary Materials.

## Supplementary Materials

The following supporting information can be downloaded at: https://www.mdpi.com/article/10.3390/toxins15090529/s1, Table S1: List of species of phytoplankton in four treatments (I, II, III, IV) during experimental exposition; Table S2: Mean concentrations of various microcystin forms and their 95% confidence intervals in experimental treatments I, II, III, and IV. Figure S1: View of microcosms; Figure S2: Dynamics of hydrochemical variables during exposure.

## References

[1] Karlson A.M.L., Duberg J., Motwani N.H., Hogfors H., Klawonn I., Ploug H., Svedén B., Garbaras A., Sundelin B., Hajdu S. (2015). Nitrogen fixation by cyanobacteria stimulates production in Baltic food webs. AMBIO.

[2] Pham T.-L., Utsumi M. (2018). An overview of the accumulation of microcystins in aquatic ecosystems. J. Environ. Manag..

[3] Sukharevich V.I., Polyak Y.M. (2020). Global occurrence of Cyanobacteria: Causes and effects (Review). Inland. Water Biol..

[4] DeMott W.R., Zhang Q.X., Carmichael W.W. (1991). Effects of toxic cyanobacteria and purified toxins on the survival and feeding of a copepod and three species of *Daphnia*. Limnol. Oceanogr..

[5] Chen L., Giesy J.P., Adamovsky O., Svirčev Z., Meriluoto J., Codd G.A., Mijovic B., Shi T., Tuo X., Li S.-C. (2021). Challenges of using blooms of *Microcystis* spp. in animal feeds: A comprehensive review of nutritional, toxicological and microbial health evaluation. Sci. Total Environ..

[6] Kaplan A., Harel M., Kaplan-Levy R.N., Hadas O., Sukenik A., Dittmann E. (2012). The languages spoken in the water body (or the biological role of cyanobacterial toxins). Front. Microbiol..

[7] Babica P., Bláha L., Maršálek B. (2006). Exploring the natural role of microcystins—A review of effects on photoautotrophic organisms. J. Phycol..

[8] Sidelev S. (2013). Influence of cyanobacterial toxins on growth of *Scenedesmus acutus* and *Gloeocapsa* sp. under laboratory conditions. Voda Khimiya i Ekologia.

[9] Leão P.N., Vasconcelos M.T., Vasconcelos V.M. (2009). Allelopathy in freshwater cyanobacteria. Crit. Rev. Microbiol..

[10] Nezbrytska I., Usenko O., Konovets I., Leontieva T., Abramiuk I., Goncharova M., Bilous O. (2022). Potential use of aquatic vascular plants to control cyanobacterial blooms: A review. Water.

[11] Hilt S., Gross E.M. (2008). Can allelopathically active submerged macrophytes stabilise clear-water states in shallow lakes?. Basic. Appl. Ecol..

[12] Blindow I., Hargeby A., Andersson G. (2002). Seasonal changes of mechanisms maintaining clear water in a shallow lake with abundant *Chara* vegetation. Aquat. Bot..

[13] Hu H., Hong Y. (2008). Algal-bloom control by allelopathy of aquatic macrophytes—A review. Front. Environ. Sci. Eng. China.

[14] Macías F.A., Galindo J.L.G., García-Díaz M.D., Galindo J.C.G. (2008). Allelopathic agents from aquatic ecosystems: Potential biopesticides models. Phytochem. Rev..

[15] Kurashov E.A., Krylova J.V., Mitrukova G.G., Chernova A.M. (2014). Low-molecular-weight metabolites of aquatic macrophytes growing on the territory of Russia and their role in hydroecosystems. Contemp. Prob Ecol..

[16] Zhu X., Dao G., Tao Y., Zhan X., Hu H. (2021). A review on control of harmful algal blooms by plant-derived allelochemicals. J. Hazard. Mater..

[17] Zhao G., Hong Y., Li L., Zhang H., Xu R., Hao Y. (2022). Selection and characterization of plant-derived alkaloids with strong antialgal inhibition: Growth inhibition selectivity and inhibitory mechanism. Harmful Algae.

[18] Wium-Andersen S. (1987). Allelopathy among aquatic plants. Arch. Hydrobiol..

[19] Mohamed Z.A. (2017). Macrophytes-cyanobacteria allelopathic interactions and their implications for water resources management—A review. Limnologica.

[20] Tan K., Huang Z., Ji R., Qiu Y., Wang Z., Liu J. (2019). A review of allelopathy on microalgae. Microbiology.

[21] Korner S., Nicklisch A. (2002). Allelopathic growth inhibition of selected phytoplankton species by submerged macrophytes. J. Phycol..

[22] Gross E.M., Erhard D., Iványi E. (2003). Allelopathic activity of *Ceratophyllum demersum* L. and *Najas marina* ssp. intermedia (Wolfgang) Casper. Hydrobiologia.

[23] Nakai S., Yoshihara T., Yamada S., Hosomi M. (2005). The Allelochemicals Accounting for the Allelopathic Effects of *Myriophyllum spicatum* on the Cyanobacterium *Microcystis aeruginosa*. http://www.regional.org.au/au/allelopathy/2005/2/1/2244_nakais.htm#TopOfPage.

[24] Nakai S., Zou G., Okuda T., Nishijima W., Hosomi M., Okada M. (2012). Polyphenols and fatty acids responsible for anti-cyanobacterial allelopathic effects of submerged macrophyte *Myriophyllum spicatum*. Water Sci. Technol..

[25] Qiming X., Haidong C., Huixian Z., Daqiang Y. (2006). Allelopathic activity of volatile substance from submerged macrophytes on *Microcystin aeruginosa*. Acta Ecol. Sin..

[26] Wang L.X., Zhang L., Zhang Y.X., Jin C.Y., Lu C.M., Wu G.R. (2006). The inhibitory effect of *Hydrilla verticillata* culture water on *Microcystic aeruginosa* and its mechanism. J. Plant Physiol. Mol. Biol..

[27] Mulderij G., Mau B., van Donk E., Gross E.M. (2007). Allelopathic activity of *Stratiotes aloides* on phytoplankton-towards identification of allelopathic substances. Hydrobiologia.

[28] Saraf M., Pandya U., Thakkar A. (2014). Role of allelochemicals in plant growth promoting rhizobacteria for biocontrol of phytopathogens. Microbiol. Res..

[29] Pakdel F.M., Sim L., Beardall J., Davis J. (2013). Allelopathic inhibition of microalgae by the freshwater stonewort, *Chara australis*, and a submerged angiosperm, *Potamogeton crispus*. Aquat. Bot..

[30] Bi Y.L., Wu S.M., Zhou S.N., Wu S.H., Xu S.J. (2019). Allelopathic effects and allelochemicals of *Myriophyllum elatinoides* on *Microcystis aeruginosa* and *Selenastrum capricornutum*. Environ. Sci..

[31] Wang H.Q., Zhang L.Y. (2017). Allelopathic activity of ethyl acetate extracts from typical emergent plants against *Microcystis aeruginosa* Kütz. Bangladesh J. Bot..

[32] Tazart Z., Douma M., Caldeira A.T., Tebaa L., Mouhri K., Loudiki M. (2020). Highlighting of the antialgal activity of organic extracts of Moroccan macrophytes: Potential use in cyanobacteria blooms control. Environ. Sci. Pollut. Res..

[33] Kurashov E., Krylova J., Protopopova E., Pereira L., Gonçalves A.M. (2021). The Use of allelochemicals of aquatic macrophytes to suppress the development of cyanobacterial “blooms”. Plankton Communities.

[34] Wang X., Yu L., Liu Y., Jiang X. (2020). Synthesis and fouling resistance of capsaicin derivatives containing amide groups. Sci. Tot. Environ..

[35] Cheng F., Cheng Z.H. (2015). Research progress on the use of plant allelopathy in agriculture and the physiological and ecological mechanisms of allelopathy. Front. Plant Sci..

[36] (2019). Nature-like and Convergent Technologies Driving the Fourth Industrial Revolution.

[37] Zhironkin S., Demchenko S., Kayachev G., Taran E., Zhironkina O. (2019). Convergent and nature-like technologies as the basis for sustainable development in the 21st century. E3S Web Conf..

[38] Sinang S.C., Daud N., Kamaruddin N., Poh K.B. (2019). Potential growth inhibition of freshwater algae by herbaceous plant extracts. Acta Ecol. Sin..

[39] Burford M.A., Gobler C.J., Hamilton D.P., Visser P.M., Lurling M., Codd G.A. (2019). Solutions for Managing Cyanobacterial Blooms: A Scientific Summary for Policy Makers.

[40] Kurashov E.A., Krylova J.V., Bataeva Y.V., Rusanov A.G., Sukhenko L.T. (2019). Algicide for Suppressing the Development of Cyanobacteria and Green Algae Based on Metabolites-Allelochemicals of Aquatic Plants. https://patents.s3.yandex.net/RU2709308C1_20191217.pdf.

[41] Kurashov E.A., Mitrukova G.G., Krylova J.V. (2018). Interannual variability of low-molecular metabolite composition in *Ceratophyllum demersum* (Ceratophyllaceae) from a Floodplain lake with a changeable trophic status. Contemp. Prob Ecol..

[42] Kurbatova S.A., Berezina N.A., Mavrin A.S., Otyukova N.G. (2022). Metabolic Rate in hydrobionts of different ecological groups in an experiment. Inland. Water Biol..

[43] Salmaso N., Tolotti M., Likens G.E. (2009). Other Phytoflagellates and Groups of Lesser Importance.

[44] Gani A., Nilawati J., Rizal A. (2015). Studi habitat dan kebiasaan makan (Food Habit) ikanronolindu (*Oryzias sarasinorum* Popta, 1905) di Danau Lindu, Sulawesi Tengah. J. Sains Dan Teknol. Tadulako.

[45] Novalina S., Diana A., Sri M., Djoko L.T., Jaya G.B.A. (2019). Food habits of the endemic rice fish (*Oryzias nigrimas*, Kottelat 1990) in Poso Lake, Central Sulawesi of Indonesia. Russ. J. Agric. Socio-Econ. Sci..

[46] Rinandha A., Andy Omar S., Tresnati J., Yanuarita D. (2023). Food habits of Matano medaka, *Oryzias matanensis* (Aurich, 1935) at Lake Towuti, South Sulawesi, Indonesia. Plant Archives.

[47] Cao X., Strojsová A., Znachor P., Zapomelová E. (2005). Detection of extracellular phosphatases in natural spring phytoplankton of a shallow eutrophic lake (Donghu, China). European J. Phycol..

[48] Salmaso N., Capelli C., Shams S., Cerasino L. (2015). Expansion of bloom-forming *Dolichospermum lemmermannii* (Nostocales, Cyanobacteria) to the deep lakes south of the Alps: Colonization patterns, driving forces and implications for water use. Harmful Algae.

[49] Capelli C., Ballot A., Cerasino L., Papini A., Salmaso N. (2017). Biogeography of bloom-forming microcystin producing and non-toxigenic populations of *Dolichospermum lemmermannii* (Cyanobacteria). Harmful Algae.

[50] Cerasino L., Capelli C., Salmaso N. (2017). A comparative study of the metabolic profiles of common nuisance cyanobacteria in southern perialpine lakes. Adv. Oceanogr. Limnol..

[51] WHO (1998). Guidelines for Drinking-Water Quality. Addendum to Volume 2: Health Criteria and Other Supporting Information.

[52] Xie L., Xie P., Guo L., Li L., Miyabara Y., Park H.D. (2005). Organ distribution and bioaccumulation of microcystins in freshwater fish at different trophic levels from the eutrophic lake Chaohu, China. Environ. Toxicol..

[53] Magalhães V.F., Marinho M.M., Domingos P., Oliveira A.C., Costa S.M., Azevedo L.O., Azevedo S.M. (2003). Microcystins (cyanobacterial hepatotoxins) bioaccumulation in fish and crustaceans from Septiba Bay (Brasil, RJ). Toxicon.

[54] Mohamed Z.A., Carmichael W.W., Hussein A.A. (2003). Estimation of microcystins in the freshwater fish *Oreochromis niloticus* in an Egyptian fish farm containing a *Microcystis* bloom. Environ. Toxicol..

[55] Wood S.A., Briggs L.R., Sprosen J., Ruck J.G., Wear R.G., Holland P.T., Bloxham M. (2006). Changes in concentration of microcystins in *Rainbow* trout, freshwater mussels, and cyanobacteria in Lakes Rotoiti and Rotoehu. Environ. Toxicol..

[56] Colas S., Duval C., Marie B. (2020). Toxicity, transfer and depuration of anatoxin-a (cyanobacterial neurotoxin) in medaka fish exposed by single-dose gavage. Aquat. Toxicol..

[57] Litvinchuk L.F., Sharov A.N., Chernova E.N., Smirnov V.V., Berezina N.A. (2023). Mutual links between microcystins-producing cyanobacteria and plankton community in clear and brown northern lakes. Food Webs.

[58] Jiang X., Yang W., Zhang L., Chen L., Niu Y. (2014). Predation and cyanobacteria jointly facilitate competitive dominance of small-bodied cladocerans. J. Plankton Res..

[59] Jiang X., Liang H., Chen Y., Xu X., Huang D. (2015). Microgeographic adaptation to toxic cyanobacteria in two aquatic grazers. Limnol. Oceanogr..

[60] Major Y., Kifle D., Niedrist G.H., Sommaruga R. (2017). An isotopic analysis of the phytoplankton–zooplankton link in a highly eutrophic tropical reservoir dominated by cyanobacteria. J. Plankton Res..

[61] Kâ S., Mendoza-Vera J.M., Bouvy M., Champalbert G., Gom-Kâ R.N., Pagano M. (2012). Can tropical freshwater zooplankton graze efficiently on cyanobacteria?. Hydrobiologia.

[62] Agasild H., Panksep K., Tõnno I., Blank K., Kõiv T., Freiberg R., Laugaste R., Jones R.I., Nõges P., Nõges T. (2019). Role of potentially toxic cyanobacteria in crustacean zooplankton diet in a eutrophic lake. Harmful Algae.

[63] Berezina N.A., Tiunov A.V., Tsurikov S.M., Kurbatova S.A., Korneva L.G., Makarova O.S., Bykova S.N. (2021). Cyanobacteria as a food source for invertebrates: Results of a model experiment. Russ. J. Ecol..

[64] Kurashov E., Kapustina L., Krylova J., Mitrukova G., Grigoryeva N. (2020). The use of fluorescence microscopy to assess the suppression of the development of cyanobacteria under the influence of allelochemicals of aquatic macrophytes. Fluorescence Methods for Investigation of Living Cells and Microorganisms.

[65] Olenina I., Hajdu S., Edler L., Andersson A., Wasmund N., Busch S., Göbel J., Gromisz S., Huseby S., Huttunen M. (2006). Biovolumes and size-classes of phytoplankton in the Baltic Sea. Baltic Sea Environment Proceedings No.106.

[66] Chernova E., Russkikh I., Voyakina E., Zhakovskaya Z. (2016). Occurrence of microcystins and anatoxin-a in eutrophic lakes of Saint Petersburg, northwestern Russia. Oceanol. Hydrobiol. Stud..

[67] Sirenko L.A., Kureishevich A.V. (1982). Determination of Chlorophyll Content in Plankton of Freshwater Reservoirs.

[68] (2000). Methods for Measuring the Mass Concentrations of Cations of Ammonium, Potassium, Sodium, Lithium, Magnesium, Strontium, Barium, and Calcium in Samples of Drinking, Natural (Including Mineral) and Waste Water Using the Kapel’ Capillary Electrophoresis System.

[69] (1999). Methods for Measuring the Mass Concentrations of Chloride Ions, Nitrite Ions, Sulfate Ions, Nitrate Ions, Fluoride Ions and Phosphate Ions in Samples of Natural, Drinking and Treated Wastewater Using the Kapel’ Capillary Electrophoresis System.

[70] (2020). Mass Concentration of Hydrocarbons and Alkalinity of Natural Waters. Measurement Procedure by Titrimetric Method.

[71] (2004). Methods for Measuring the Mass Concentrations of the Total Nitrogen in Natural and Treated Wastewater by the Titrimetric Method.

[72] (1997). Methods for Measuring the Mass Concentration of Total Phosphorus in Samples of Natural and Treated Wastewater by the Photometric Method after Oxidation with Persulfate.

[73] (1997). Methods for Measuring the Content of Dissolved Oxygen in Samples of Natural and Treated Wastewater by the Iodometric Method.

