# Supplementary material for: Effects of Algicidal Macrophyte Metabolites on Cyanobacteria, Microcystins, Other Plankton, and Fish in Microcosms
Source: Toxins (Basel). 2023 Aug 28;15(9):529. doi: 10.3390/toxins15090529 (PMC10535574; doi:10.3390/toxins15090529)
Supplement: Supplementary file 1 [file toxins-15-00529-s001.zip › toxins-2486350-supplementary.pdf]

toxins

Article

Effects of Algicidal Macrophyte Metabolites on Cyanobacteria, Microcystins, Other Plankton, and Fish in Microcosms

Supplementary materials

Figure S1: View of microcosms setup. Photo A. Mavrin.

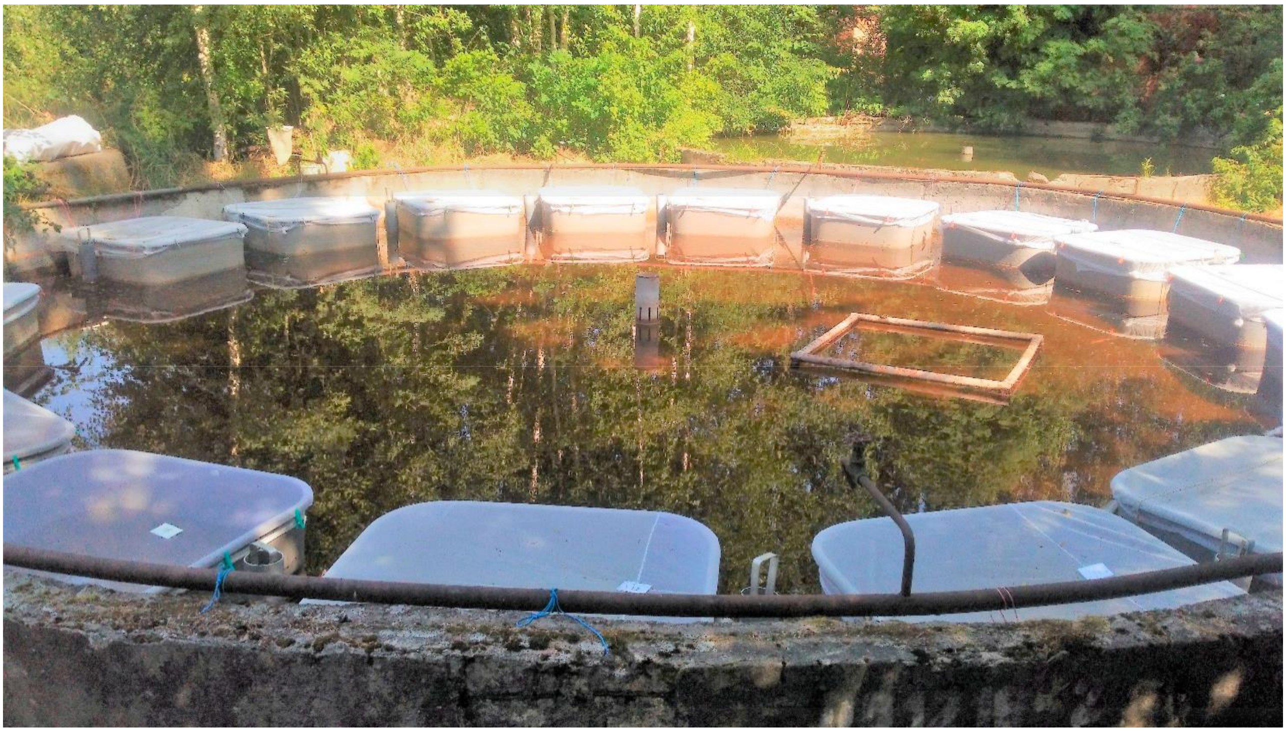

Figure S2: Dynamics of hydrochemical variables during exposure. Mean values and confidence intervals are given (n=3).

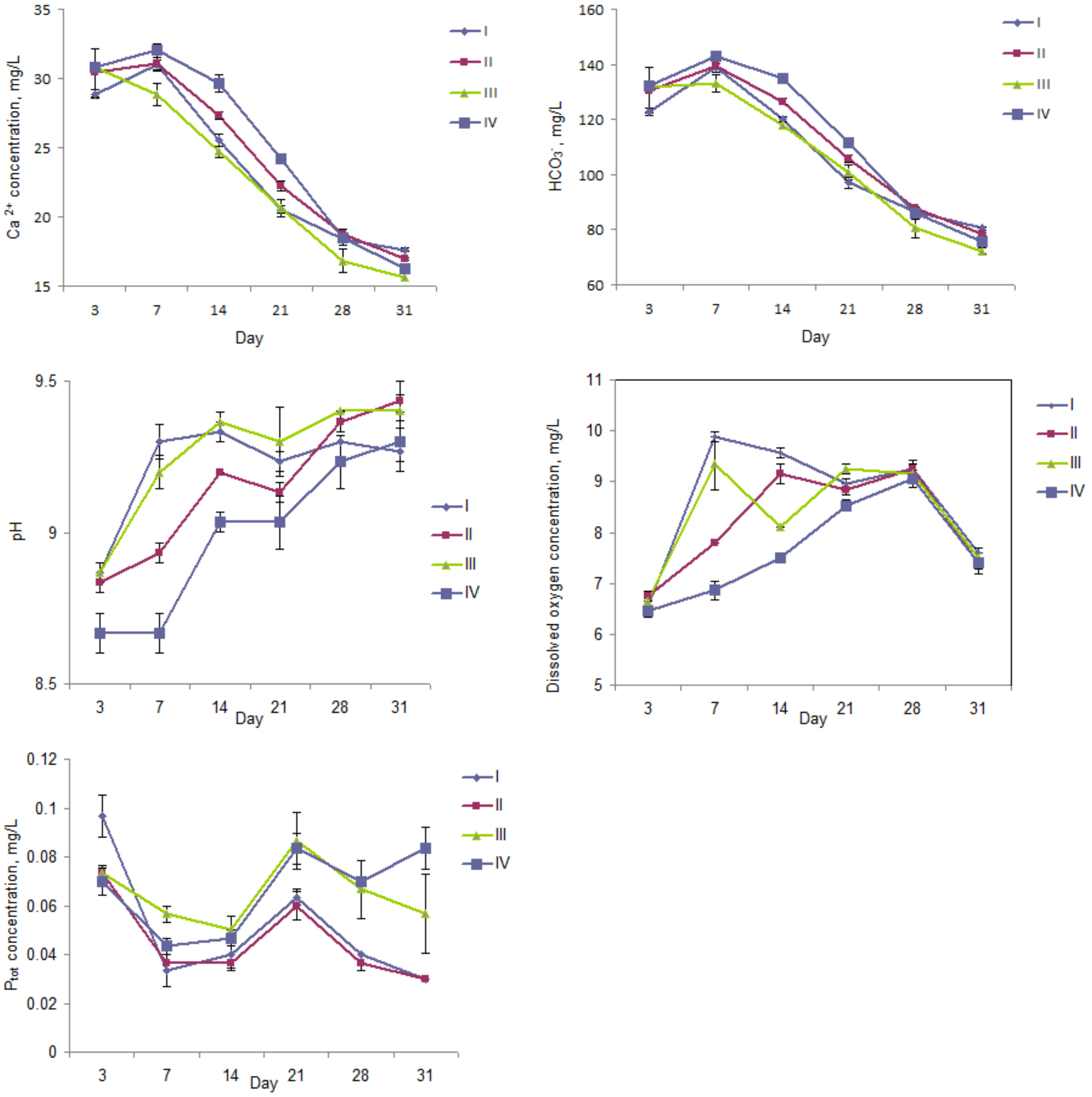

Table S1. List of species of phytoplankton in four treatments (I, II, III, IV) during experimental exposition.

	Treatment			
	**I**	**II**	**III**	**IV**
**Cyanobacteria**				
Dolichospermum spiralis	xxx	xxx	xxx	xxx
Dolichospermum affinis	x	x	x	x
Pseudanabaena limnetica	x	x		
Aphanocapsa delicatissima	x	x	x	x
Aphanocapsa planctonica	x	x	xx	x
Aphanothece stagnina	x	-	-	-
Aphanizomenon flos-aquae	xxx	xxx	xxx	xxx
Aphanizomenon issatschenkoi	x	x	xx	xx
Aphanizomenon gracile	-	-	xx	-
Coelosphaerium kutzingianum	x	-	x	x
Nostoc kihlmani	-	-	-	x
Phormidium sp.	x	-	-	-
Pseudoanabaena sp.	x	-	x	-
Microcystis aeruginosa	xx	x	xx	xxx
Planktolyngbya limnetica	x	xx	x	xx
Merismopedia sp.	x	x	x	x
Nodularia sp.	x	x	x	x
Snowella fenica	x	x	x	x
Synechocystis sp.	-	-	x	x
**Cryptophyta**				
Komma caudata	x	x	x	x
Cryptomonas sp.	xx	xxx	xx	x
Cryptomonas marsonii	xxx	xxx	xx	x
Chroomonas acuta	x	x	x	x
Katablepharis ovalis	x	-	x	-
**Chrysophyta**				
Dynobrion cilindricum	-	-	-	x
Mallomonas sp.	x	x	x	x
**Dynophyta**				
Ceratium hirundinella	x	xx	xx	x
Gymnodinium sp.	x	x	x	x
Peridinium aciculiferum	-	-	x	x
Peridinium sp.	-	x	x	x
**Bacillariophyta**				
Aulacoseira granulata	xxx	xxx	xxx	xxx
Fragilaria sp.	x	x	x	x
Navicula sp.	-	-	x	-
Nitzchia acicularis	x	x	x	x

Pinularia sp.	x	-	-	-
Synedra nana	x	x	x	x
Belonastrum berolinensis	x	x	x	-
Ulnaria acuc	-	x	xx	x
**Chlorophyta**				
Chlorella sp.	x	x	x	x
Chlamydocapsa planctonica	-	-	x	-
Coelastrum spharicum	x	-	-	-
Coelastrum microporum	x	x	x	x
Coelastrum astroideun	x	x	x	x
Botryococcus braunii	x	x	x	x
Actinastrum sp.	-	-	-	x
Ankyra judayi	-	x	x	-
Ankyra ancora	x	-	-	x
Ankistrodesmus fusiformis	xx	x	x	xx
Dictyosphaerium ehrenberg	--	x		x
Dictyosphaerium subsolitariuu	x	x	x	x
Dictyosphaerium pulhellum	-	x		x
Geminella planctonica	-	x	x	-
Geminella pulcha	-	x	-	-
Gloeotila pelagica	-	x	x	x
Gloeotila fenica	x	x	-	x
Keratococuus suecicus	-	-	-	x
Korschikoviella limnetica	x	x	x	-
Kirchneriella obesa	-	-	-	x
Kirchneriella lynaris	-	-	-	x
Pediastrum borianum	x	x	x	x
Planctonema lauterbornii	x	x	x	x
Planktosphaeria gelatinosa	x	x	-	-
Pandorina morum	x	-	-	x
Eudorina elegans	-	-	-	x
Sphaerocystis schroeteri	x	x	x	x
Oocystis lacustris	x	x	x	x
Monoraphidium contortum	x	-	x	x
Monoraphidium dubovskii	x	x	-	x
Monoraphidium mirabile	x	-	x	x
Monoraphidium griffithii	x	x	x	x
Nephrocytium agardhianum	-	x	x	x
Volvox sp.	x	x	-	-
Scenedesmus quadricauda	x	x	x	x
Scenedesmus acuminatus	-	x		x
Scenedesmus sempervirens	x	x	x	x

Selenastrum gracile	x	-	-	-
Tetraëdron minimum	x	x	x	x
Sorastrum spinulosum	-	x	-	x
**Charophyta**				
Mougeotia sp.	xx	xxx	xxx	xxx
Elakatothrix	x	x	x	x
Cosmarium meneghinii	x	x	x	x
Cosmarium impressulum	x	-	-	x
Closterium acutum	-	x	x	x
Spirogyra sp.	-	x	-	-
Spondylosium planum	-	-	x	-
Staurastrum	x	x	x	x
Staurodesmus	x	-	-	-

Note.

xxx species dominates, accounting for more than 10% of the total phytoplankton biomass

xx species less than 5% of total biomass

x species makes up 5 to 10% of the total biomass

- species not found.

Table S2. Mean concentrations of various microcystin forms and their 95% confidence intervals in experimental treatments I, II, III, and IV. The different letters show significant differences (p < 0.05) between pairs per date.

Treatment	I	II	III	IV	I	II	III	IV	I	II	III	IV
Mean	19.07	19.07	19.07	19.07	5.08	5.08	5.08	5.08	19.08	19.08	19.08	19.08
[D-Asp3]MC-LR	0.338	0.309	0.409	0.433	0.316	0.093	0.437	0.363	0.222	0.037	0.454	0.491
MC-LR	6.405	6.744	9.371	9.360	0.280	0.401	2.326	2.043	0.123	0.143	0.683	1.248
[D-Glu-OCH36]MC-LR	0.092	0.101	0.140	0.151	< 0.001	< 0.001	0.024	0.021	< 0.001	< 0.001	< 0.001	< 0.001
[D-Asp3]MC-RR	0.006	0.010	0.014	0.012	< 0.001	< 0.001	0.000	0.002	< 0.001	< 0.001	< 0.001	< 0.001
MC-LW	0.020	0.022	0.035	0.041	< 0.001	0.002	0.013	0.009	< 0.001	< 0.001	< 0.001	< 0.001
[Dha7]MC-YR	0.532	0.534	0.617	0.699	0.516	0.128	0.465	0.367	0.208	0.065	0.238	0.260
MC-RR	0.039	0.050	0.075	0.076	0.001	0.002	0.006	0.005	0.003	0.014	0.007	0.011
MC-YR	1.911	1.988	1.639	1.609	3.635	0.658	5.229	2.624	1.756	0.247	5.694	4.028

Treatment	I	II	III	IV	I	II	III	IV	I	II	III	IV
95% confidence intervals	19.070	19.070	19.070	19.070	5.080	5.080	5.080	5.080	19.080	19.080	19.080	19.080
[D-Asp3]MC-LR	0.026	0.022	0.336	0.072	0.086	0.031	0.044	0.030	0.011	0.000	0.056	0.017
MC-LR	0.494	0.020	0.101	1.120	0.109	0.048	0.952	0.194	0.033	0.091	0.252	0.043
[D-Glu-OCH36]MC-LR	0.007	0.007	0.115	0.025	-	-	0.002	0.002	-	-	-	-
[D-Asp3]MC-RR	0.000	0.000	0.000	0.002	-	-	0.001	0.000	-	-	-	-
MC-LW	0.002	0.002	0.029	0.007	-	0.001	0.001	0.001	-	-	-	-
[Dha7]MC-YR	0.006	0.031	0.220	0.016	0.006	0.001	0.008	0.010	0.007	0.000	0.004	0.003
MC-RR	0.003	0.004	0.062	0.013	0.000	0.001	0.001	0.000	0.000	0.000	0.001	0.000
MC-YR	0.147	0.260	1.444	0.330	0.180	0.083	0.167	0.437	0.274	0.016	0.324	0.140

Treatment	I	II	III	IV	I	II	III	IV	I	II	III	IV
Letter of significance	19.07	19.07	19.07	19.07	5.08	5.08	5.08	5.08	19.08	19.08	19.08	19.08
[D-Asp3]MC-LR	a	a	a	a	b	a	b	b	b	a	c	c
MC-LR	a	a	a	a	a	a	b	b	a	a	b	c
[D-Glu-OCH36]MC-LR	a	a	b	c	-	-	a	a	-	-	-	-
[D-Asp3]MC-RR	a	ab	b	b	-	-	a	a	-	-	-	-
MC-LW	a	a	b	b	-	a	b	b	-	-	-	-
[Dha7]MC-YR	a	a	a	a	c	a	c	b	a	b	a	a
MC-RR	a	b	c	c	a	a	b	b	a	d	b	c
MC-YR	a	a	a	a	b	a	c	b	b	a	c	c
